# Dedifferentiated Paratesticular Liposarcoma with Osseous Metaplasia

**DOI:** 10.1155/2015/965876

**Published:** 2015-04-16

**Authors:** Kostas Chondros, Ioannis Heretis, Michael Papadakis, Vasiliki Bozionelou, Emmanouil Mavromanolakis, Nikolaos Chondros, Charalampos Mamoulakis

**Affiliations:** ^1^Department of Urology, University General Hospital of Heraklion, University of Crete Medical School, Heraklion, 71110 Crete, Greece; ^2^Department of Pathology, University General Hospital of Heraklion, University of Crete Medical School, Heraklion, 71110 Crete, Greece; ^3^Department of Medical Oncology, University General Hospital of Heraklion, University of Crete Medical School, Heraklion, 71110 Crete, Greece

## Abstract

Paratesticular liposarcoma is a rare tumour of the genitourinary track but the most common of all sarcomas in adults. The dedifferentiated variation occurs only in 10% of liposarcoma cases. The typical clinical presentation is similar to an inguinal hernia or a benign lipoma. We present the case of a dedifferentiated paratesticular liposarcoma with osseous metaplasia of the spermatic cord, in a male presented with acute scrotum.

## 1. Introduction

Liposarcoma is a malignant neoplasm of the adipose tissue representing the second most common type of sarcoma following malignant fibrous histiocytoma (20% of the cases) [1]. It is the most common type of genitourinary sarcomas in adults, and it is usually located in the spermatic cord, epididymis, or testis. Histologic classification has been well established by the World Health Organization (WHO 2013) and includes four different subtypes of liposarcoma among which the well-differentiated liposarcoma (WDL) represents the largest subgroup (40–45%) [2]. Cell dedifferentiation, as a pathological process, confers a poorer prognosis and occurs in up to 10% of WDL. Different pathological components such as leiomyosarcomatous differentiation, osteosarcomatous differentiation, and bone formation have been described in case reports with a potential role in the overall prognosis [3–6]. The clinical presentation of genitourinary liposarcomas is that of a painless, palpable large unilateral scrotal or inguinal mass. A differential diagnostic problem emerges due to similar symptomatology with inguinal hernias or subcutaneous lipomas. Radiological evaluation is helpful for the diagnosis. Other clinical presentations, such as acute scrotum, are rather rare and an immediate surgical exploration is required.

## 2. Case Presentation

**Figure 1:**
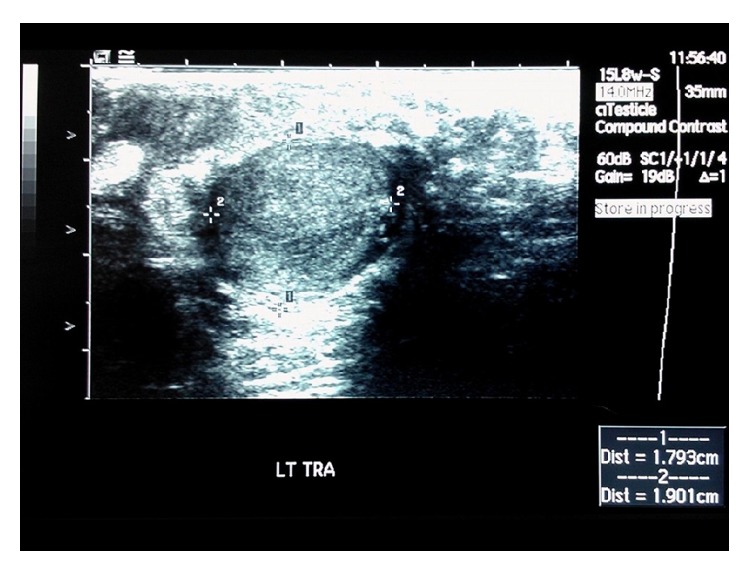
Scrotal US scan at the emergency department. Right-sided solid paratesticular mass.

**Figure 2:**
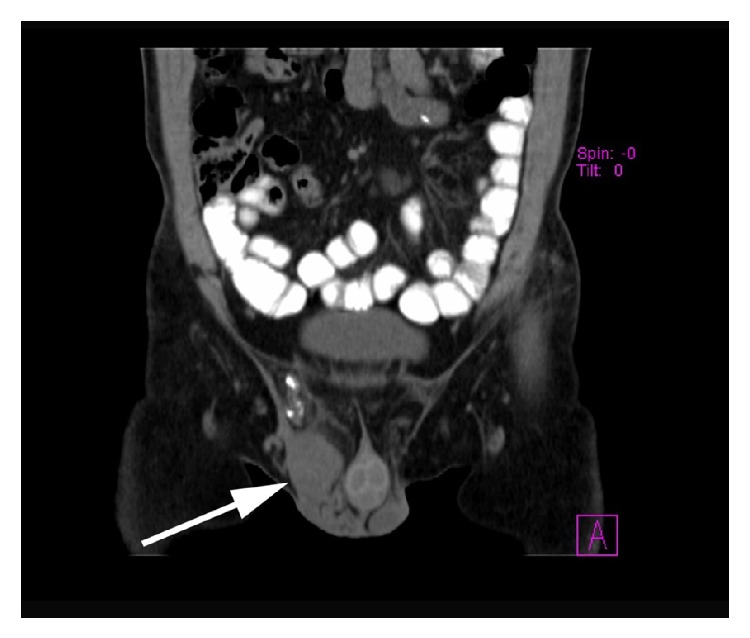
CT scan showing a solid soft-tissue mass of 3.6 × 3.7 × 5.8 cm in the right scrotum (white arrow).

**Figure 3:**
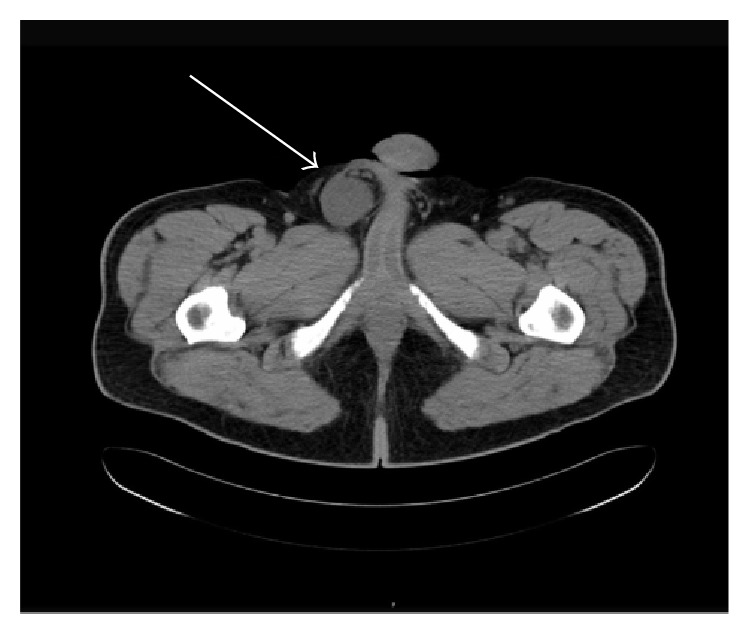
CT scan showing the solid soft-tissue paratesticular mass in contact with the right spermatic cord (white arrow).

A 61-year-old male presented to the emergency department with acute right inguinal pain with concomitant ipsilateral scrotal swelling over the past 24 hours. Clinical examination revealed a painful solid mass on the right spermatic cord and normal testicles. The patient was afebrile, without any lower urinary tract symptoms or signs of urinary infection. Laboratory investigation was normal including testicular tumor markers (serum levels of *α*-fetoprotein (AFP), human chorionic gonadotropin (hCG), and lactate dehydrogenase (LDH)) within normal limits. Radiological evaluation included scrotal ultrasonography (US) and computed tomography (CT) scan. US scan revealed a right-sided solid paratesticular mass with normal testes (Figure 1). The CT scan documented the presence of a solid soft-tissue mass 3.6 × 3.7 × 5.8 cm originating from the right spermatic cord and a small calcification inside the inguinal canal. Retroperitoneal and pelvic lymph nodes were within normal limits (Figures 2 and 3).

**Figure 4:**
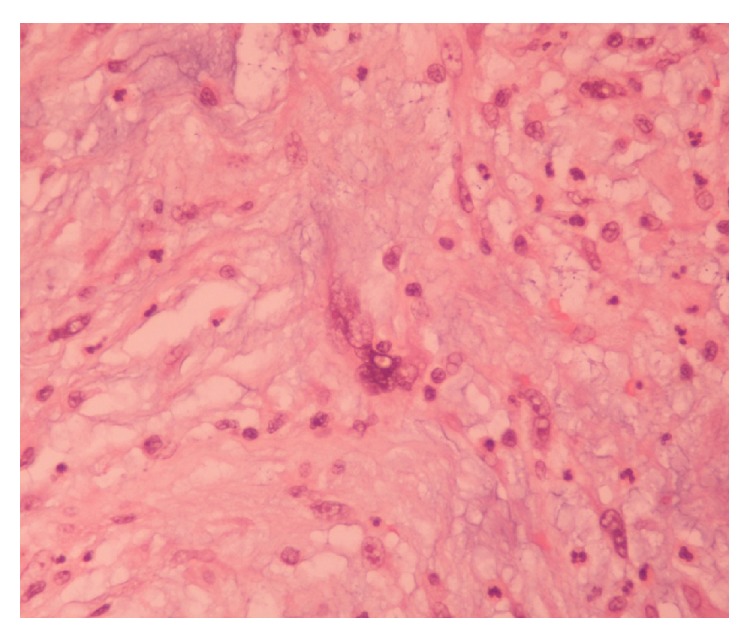
Anaplastic spindle cell neoplasm with prominent myxoid and collagenous stroma with a high degree nuclear pleomorphism. The diagnosis of low grade DDL was made after immunohistochemical confirmation. Hematoxylin-eosin stain (H&E) ×400.

**Figure 5:**
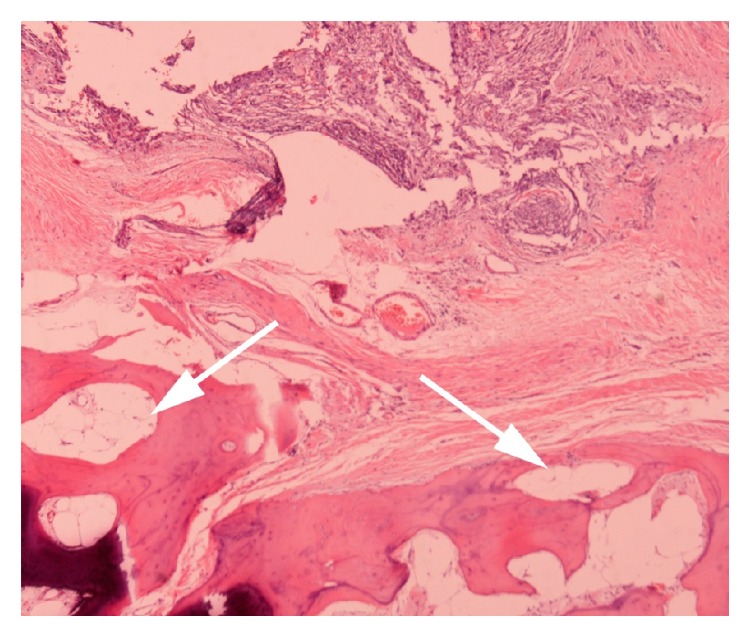
Presence of mature bone formation (white arrows) (H&E) ×400.

Due to the progressive severe hemiscrotal pain the patient was submitted to an emergency diagnostic scrotal exploration and surgical removal of the mass. The pathological investigation revealed a variably anaplastic spindle cell neoplasm with prominent and inflamed collagenous stroma and a high degree of cellular atypia. Immunohistochemical markers of the tumor were as follows: S-100 (−), HHF-35 (−), vimentin (+), C-kit (−), desmin (+), SMA (+), and CD 34 (+). Cytogenetic evaluation in substains showed strong and diffuse nuclear positivity for murine double minute 2 (MDM2) and cyclin-dependent kinase-4 (CDK4) consistent with translocation of these genes on the long arm of chromosome 12. These findings were suggestive of a low grade dedifferentiated liposarcoma (DDL) (Figure 4). A supplementary radical orchiectomy with high ligation of the spermatic cord was immediately performed due to positive surgical margins. In the second surgical specimen, mature bone formation within the spermatic cord was detected due to osseous metaplasia of the actual neoplasm (Figure 5). Surgical margins were confirmed this time to be negative and the testicle showed mild atrophy without any other significant anomalies.

Due to high comorbidity (coronary artery disease, history of myocardial infraction with angioplasty and stent placement, and hypertension), the patient was not submitted to adjuvant chemotherapy as decided in our multidisciplinary tumor board. Additionally, the option of local radiotherapy was not considered beneficial. Therefore an intensive follow-up schedule was applied, with chest/abdomen CT scans every 3 months for the first year and every 6 months for the second year. Two years after the initial diagnosis there is no local or systematic recurrence.

## 3. Discussion

Liposarcomas are classified according to WHO classification of soft-tissue tumors (2013) in four major subtypes: atypical lipomatous tumor/WDL, DDL, myxoid liposarcoma, and pleomorphic liposarcoma [2]. Histological type has been showed in several studies to be an important prognostic factor for the recurrence and disease-specific mortality [4–7]. Dedifferentiation is detected in up to 10% of WDL of any subtype and it is related to poorer prognosis. However, it presents less aggressive clinical behavior compared to other high grade pleomorphic sarcomas [8]. Histopathologically, there are two grades (low and high) of DDL. Low grade dedifferentiation is characterized most often by the presence of uniform fibroblastic spindle cells with mild nuclear atypia. Cytogenetics showed similar positiveness of MDM2, TP53 and CDK4, and chromosome translocations (12q14-15), with WDL. Heterologous dedifferentiation may occur in about 5–10% of the cases as well as osseous metaplasia, which has not been related with aggressiveness or invasiveness of the tumor [9].

The clinical presentation of DDL typically includes a large painless mass, which may be incidentally detected (particularly in the retroperitoneum). Genitourinary localization of DDL has been scarcely described in the literature while other subtypes represent the most common diagnosis of genitourinary sarcomas, in adults. Occasionally, scrotal pain or discomfort can occur due to rapid mass enlargement or infection [10]. CT or Magnetic Resonance Imaging (MRI) scan is helpful to differentially diagnose a solid palpable inguinal or scrotal mass from a hernia or other potential cystic lesions of the epididymis and detect any positive pelvic or retroperitoneal lymph nodes and/or distant metastases [11].

A pretreatment surgical or US-guided needle biopsy is also indicated to diagnose the type and the grade of the sarcoma. Paratesticular sarcomas should be treated by radical orchiectomy with high ligation of the spermatic cord [12]. In the absence of radiological findings compatible with metastasis, patients with liposarcomas should not undergo any retroperitoneal lymph node dissection or biopsy, due to the relevant metastatic risk (15–20%) at the presence and the fact that the vast majority of patients have localized disease at diagnosis. Contrawise, high local recurrence rates have been reported up to 90% and were correlated with the subtype, the size of the initial tumor, and the surgical margins state [13]. Optimally, radical resection with negative margins ≥1 cm is possibly curative.

External adjuvant radiation therapy is a controversial option. There are several studies published regarding combination therapy, in large retroperitoneal liposarcomas and the results were beneficial for the patients. Other studies reported that the administration of adjuvant radiotherapy did not seem to confer survival benefit. As far as paratesticular liposarcoma is concerned the data are limited. The trend is that adjuvant radiotherapy should be used in patients with positive surgical margins or when the adequacy of local excision is in doubt. In these cases there is a decrease in local recurrence up to 44% but there is no impact on the overall survival rate at 5 years [14]. Definitive radiation therapy is recommended for patients who are not surgical candidates, with radiation doses range from 60 to 70 Gy.

Systemic chemotherapy should be given to patients with evidence of retroperitoneal or distant metastases. A variety of chemotherapeutic agents and combinations are available based mainly on doxorubicin, ifosfamide, mesna, and dacarbazine [15]. Limited data are available for adjuvant chemotherapy in paratesticular liposarcoma. Age, performance status, size, grade, location, type of initial surgery, and margin status should be taken into account when there is a consideration for adjuvant therapy. Combination chemotherapy regimens with concurrent radiation therapy have higher response and prohibitive toxicities and therefore should only be applied in selected patients [16].

There are several survival rates reported, depending on the histologic subtype, grade, size, surgical margins status, and the primary site of liposarcomas. Five-year survival rates of paratesticular liposarcomas range from 20% to 80% while the long-term survival of men with all paratesticular sarcomas is approximately 50%. Dedifferentiation is mainly considered to be a poor prognostic factor especially for local recurrence whereas well-differentiated recurrences may be noted. Low grade is not a prognostic factor in these types and metastatic risk is up to 20% [17].

An intensive follow-up schedule is worthwhile for these patients. Radiologic evaluation with CT or MRI is highly recommended at least every 3–6 months for the first 3 years and annually thereafter [15].

In our case, the patient had a low grade paratesticular DDL that was surgically excised with negative surgical margins. The osseous metaplasia was not considered to confer any aggressiveness to the tumor. The radiologic evaluation was negative for lymph node disease or distant metastasis. The patient had significant comorbidities and medium performance status and thus no adjuvant therapy was applied.

## 4. Conclusion

Paratesticular liposarcoma requires aggressive surgical approach for curative treatment with radical orchiectomy. The dedifferentiated subtype is less common with a trend to recur locally. Osseous metaplasia is rare and does not represent an unfavorable prognostic factor. However, these tumors, due to their potential metastatic risk, should be treated with radical orchiectomy followed by additional therapy in cases of local recurrence or distant metastases and the patients should undergo intensive radiologic follow-up.
